# Effects of total parenteral nutrition and electrolyte solutions with or without glutamine on the hormonal profiles of horses subjected to exploratory laparotomy

**DOI:** 10.29374/2527-2179.bjvm005725

**Published:** 2026-01-14

**Authors:** Cintia Ferreira, Maristela Silveira Palhares, Ubiratan Pereira de Melo, Fabíola de Oliveira Paes Leme, Renata de Pino Albuquerque Maranhão, Hélio Chiarini Garcia, Valentim Arabicano Gheller

**Affiliations:** 1 Centro Universitário Maurício de Nassau (Uninassau). Natal, RN, Brazil.; 2 Programa de Pós-Graduação em Ciência Animal, Departamento Clínica e Cirurgia Veterinárias. Escola de Veterinária, Universidade Federal de Minas Gerais. Pampulha, MG. Brazil.; 3 Instituto de Ciências Biológicas, Universidade Federal de Minas Gerais. Pampulha, MG. Brazil.

**Keywords:** horse, starvation, ghrelin, insulin, leptin, cavalo, inanição, grelina, insulina, leptina

## Abstract

Starvation and nutritional support clinically influence horses, particularly during the treatment of gastrointestinal diseases and post-surgical recovery. In this study, we aimed to evaluate the effects of different nutritional strategies on the endocrine response of horses subjected to exploratory laparotomy followed by food deprivation. Sixteen healthy adult horses were randomly allocated into four groups (n=4) based on treatments: enteral fluid therapy without (ENTFL) or with glutamine (ENTGL), and parenteral fluid therapy with glucose (PARFL) or total parenteral nutrition with glutamine (PARGL). Treatments were administered for 144 hours of starvation followed by 144 hours of refeeding. Blood samples were collected at five time points for hormonal (leptin, ghrelin, adiponectin, cortisol, insulin, free T3, and free T4) analyses. Serum insulin and glucose significantly varied across all groups, with the PARGL group exhibiting hyperinsulinemia and persistent hyperglycemia during starvation. No significant alterations in free T3 or T4 concentrations were observed, which is potentially attributed to elevated serum glucose levels maintaining thyroid hormone stability. Notably, cortisol levels increased in the ENTGL group 72 hours after the onset of starvation. Ghrelin significantly increased only in the PARGL group during starvation, and it decreased after refeeding. Adiponectin levels were enhanced in the PARFL group during deprivation, which dropped after refeeding. Starvation reduced leptin levels in the ENTGL and PARGL groups, which were increased after refeeding. Despite hormonal changes, enteral nutrition maintained glycemic control, indicating stable intestinal absorptive function. These findings highlight the influence of nutritional route and composition, especially glutamine and glucose, on endocrine adaptation during starvation and refeeding. However, limitations such as the small sample size and absence of an absolute fasting group constrain the interpretations. Future studies involving broader hormonal panels and longer follow-up can better clarify neuroendocrine regulation during nutritional stress in horses.

## Resumo

A inanição e o suporte nutricional são aspectos clínicos relevantes na medicina equina, especialmente durante o manejo de doenças gastrointestinais e na recuperação pós-operatória. Este estudo teve como objetivo avaliar os efeitos de diferentes estratégias nutricionais sobre a resposta endócrina de equinos submetidos à privação alimentar após laparotomia exploratória. Dezesseis cavalos adultos saudáveis foram distribuídos aleatoriamente em quatro grupos (n=4): hidratação enteral sem (ENTFL) ou com glutamina (ENTGL), e hidratação parenteral com glicose (PARFL) ou nutrição parenteral total com glutamina (PARGL). Os tratamentos foram administrados durante 144 horas de inanição seguidas de 144 horas de realimentação. Amostras sanguíneas foram coletadas em cinco momentos para análise hormonal (leptina, grelina, adiponectina, cortisol, insulina, T3 livre e T4 livre). Foram observadas variações significativas nas concentrações séricas de insulina e glicose em todos os grupos, com hiperinsulinemia e hiperglicemia persistente no grupo PARGL durante a fase de inanição. Não foram identificadas alterações significativas nos níveis de T3 e T4 livres, possivelmente devido à manutenção da glicemia elevada. Os níveis de cortisol aumentaram 72 horas após o início da inanição, especialmente no grupo ENTGL. A grelina aumentou apenas no grupo PARGL durante a inanição e reduziu após a realimentação. A adiponectina aumentou no grupo PARFL na privação e reduziu após a realimentação. A leptina diminuiu nos grupos ENTGL e PARGL durante a inanição e aumentou após a realimentação. Apesar das alterações hormonais, a via enteral manteve o controle glicêmico, indicando preservação da capacidade absortiva intestinal. Este estudo destaca a influência da via e composição nutricional, especialmente a presença de glutamina e glicose, na adaptação endócrina durante a privação e realimentação. Limitações como o número reduzido de animais e a ausência de um grupo em jejum absoluto restringem a interpretação dos dados. Estudos futuros devem ampliar o painel hormonal e o tempo de acompanhamento para melhor compreensão da regulação neuroendócrina em situações de estresse nutricional em equinos.

## Introduction

In the equine species, a variable period of starvation is recommended in cases of abdominal disease (Carr, 2018) and in the pre- and postoperative periods to minimize the risk of complications such as postoperative colic (Bailey et al., 2016). However, food withdrawal is not a physiological practice, owing to the food intake pattern of horses, which are monogastric herbivores physiologically adapted for digesting large amounts of high-fiber forages. Moreover, under natural conditions, grazing animals typically spend 12–18 h per day foraging on pasture, with a duration of 2–3 h for each meal, separated by short breaks for digestion, locomotion, and other social activities (Maia et al., 2017; Melo et al., 2021).

Decreased appetite and/or inability to eat, which are associated with different conditions, result in malnutrition (Carr, 2018; Di Filippo et al., 2021). Enteral or parenteral nutritional support presents a preventive and therapeutic strategy for malnutrition; it has become increasingly important in managing the response to clinical–surgical treatments, promoting tissue repair, and improving immune function (Bæk et al., 2020; Lawson et al., 2021). Nutritional support should be considered for horses with increased metabolic rates, such as growing animals, horses with a history of malnutrition or hypophagia, patients with metabolic disorders, and those with diseases that result in an increased metabolic rate (Melo et al., 2008; Carr, 2018; Melo et al., 2025).

Parenteral nutrition is often required for horses that are anorectic for more than 12–24 h, and sooner in horses predisposed to hypertriglyceridemia and those with poor body reserves (Magdesian & Bozorgmanesh, 2018). The use of parenteral nutrition in horses has been rarely explored; however, it has been described in conference proceedings, individual case reports, retrospective studies, and review articles (McKenzie, 2015). Recently, Melo et al. (2023, 2025) provided new insights into the use of parenteral nutrition, contributing to the understanding and clinical application of this therapeutic approach in equine medicine. Parenteral nutrition can ensure adequate nutrient supply, particularly during critical illness or the post-surgical recovery phase. However, several veterinarians reluctantly implement it, considering the complexities of formulation, administration, and clinical monitoring as well as the potential complications and expenses experienced by clients (McKenzie, 2015).

The classic profile of the hormonal response to starvation includes increased plasma concentrations of glucocorticoids, catecholamines, ghrelin, glucagon, and growth hormone, and reduced concentrations of insulin, gonadotropins, leptin, and thyroid hormones. These hormonal responses serve as an afferent stimulus for the hypothalamic response to starvation, resulting in reduced energy expenditure and metabolism (McCue, 2010; Melo et al., 2011). Nutritional support during periods of convalescence can minimize or inhibit these hormonal changes.

Hence, in this study, we aim to evaluate the effects of total parenteral nutrition associated with glutamine, enteral fluid therapy with or without glutamine, and parenteral fluid therapy associated with glucose on the hormonal profiles of horses under starvation after exploratory laparotomy.

## Material and methods

Sixteen healthy male and female adult horses of mixed breed, aged between 4 and 14 years, with a mean body weight (BW) of 248.40 ± 2.28 kg and a body score index of 3–4 (scale of 1–5) were included in this study. These horses were divided into four groups, with four horses in each group. The horses that received endo- and ectoparasiticide were housed in paddocks and fed daily with commercial feed (1 kg/100 kg BW), Tifton hay (1 kg/100 kg BW), chopped elephant grass (*Pennisetum purpureum*), water, and mineral salt *ad libitum*. After 30 days of adaptation, they were randomly divided into four experimental groups following a previously described method (Melo et al., 2021).

The animals of the ENTFL group were administered an enteral electrolyte solution containing 5.7 g NaCl, 3.78 g NaHCO3, 0.37 g KCl, and 10 g glucose, diluted in 1,000 mL of water, via the nasogastric route. The horses in the ENTGL group received enteral fluid therapy with electrolytes (5.7 g NaCl, 3.78 g NaHCO_3_, 0.37 g KCl, and 10 g glucose) associated with glutamine (L-glutamine, Ajinomoto do Brasil Indústria e Comércio de Alimentos Ltda, Laranjal Paulista/SP, Brazil).

A maintenance rate of 60 ml/kg was considered while calculating the total volume of fluid administered to the ENTF and ENTGL groups over 24 hours (Melo et al., 2010). The calculated total volume of fluid was divided by 12 to decide the volume administered in bolus every 2 h using an 11 mm × 16 mm nasogastric tube. Glutamine (ENTGL) was administered at a dose of 0.5 g/kg BW. The calculated total amount of glutamine was divided by 12 to calculate the volume administered in a diluted form in enteral fluid therapy every 2 h. The volume of fluid administered (ENTFL and ENTGL groups) and the amount of glutamine (ENTGL group) were adjusted daily according to BW.

The horses in the PARGL group received TPN associated with glutamine. To prepare TPN, equal amounts of amino acid solutions (10% Aminoven, Fresenius Kabi, Barueri/SP, Brazil) were mixed with lipids (20% Lipovenos, Fresenius Kabi) and 50% glucose (Fresenius Kabi) (33.33% amino acids, 33.33% lipids, and 33.33% of 50% glucose) to meet the energy requirements for daily maintenance. Sterile glutamine solution (1.5%, 15 g/L) was intravenously administered at a dose of 0.5 g/kg BW. The concentration of the solution was maintained at 15 g/L, and only the final infused volume was adjusted according to BW to provide the target daily dose of 0.5 g/kg BW.

The total amounts of TPN and glutamine administered were daily adjusted based on the body weight; both solutions were continuous infusions via venous access into the right external jugular vein. The rate of administration (ml/h) was calculated by dividing the total infused volume by 24. The appropriate amount of intravenous fluid therapy with lactated Ringer’s solution was administered for daily maintenance.

The PARFL group was treated with parenteral fluid, administered at a dose of 60 ml/kg BW. Half of the fluid was administered through lactated Ringer’s solution, and the other half was provided via a saline solution (0.9% NaCl). Subsequently, 50% glucose diluted with a saline solution was administered at a dose of 1.5 g/kg BW. Both solutions were administered as continuous infusions via venous access into the right external jugular vein.

For the infusion of TPN (PARGL group) and parenteral fluid therapy (PARFL group), venous access was obtained using a double-lumen catheter (Duocath – 12F, Intra Special Catheters, Germany) with a coaxial size of 12 gauge and a length of 20 cm.

The present study was conducted in two distinct phases, each phase lasting for 144 hours: In Phase 1, treatments were administered according to the experimental groups following exploratory laparotomy. During this phase, the horses in different groups were starved, receiving only the specific water and the corresponding nutritional resources. In Phase 2, the feed was gradually reintroduced. On days 1 and 2 of Phase 1, total parenteral nutrition (TPN) administered to the PARGL group provided 50% and 75%, respectively, of the maintenance energy requirements; TPN provided 100% of these energy demands from the third to the last day of Phase 1. In Phase 2, Tifton hay, commercial concentrate, and water were gradually reintroduced to horses. On the first day, 4 kg of Tifton hay, divided into two meals of 2 kg each, was offered 12 hours apart, which was supplemented with 0.5 kg of commercial concentrate. On the second day, the amount of hay was increased to 8 kg, which was provided in two meals of 4 kg each; moreover, 1 kg of concentrate was included. From the third day onward, the feeding regimen was adjusted to the levels provided during the pre-experimental adaptation period.

To evaluate the effect of starvation and nutritional support on the morphological characteristics of the gastrointestinal mucosa during the starvation and refeeding phases, intestinal biopsies were obtained from the small and large intestines via laparotomy through the flank of the animals in the station following the method described by Ferreira et al. (2022) and Melo et al. (2022). After the first laparotomy, anti-inflammatory and systemic antibiotic therapy were performed via intravenous (IV) administration of 4.4 mg/kg phenylbutazone, once daily (SID) for 10 days, and intramuscular (IM) injection of benzathine Penicillin G 25.000 IU/kg, SID, for 10 days. Postoperative care included assessment of pain or post-surgical complications, including hemorrhage and infection, in addition to dressings at the incision site as described by Melo et al. (2022).

To assess the hormonal profile, blood samples were obtained through central venipuncture of the external jugular vein using vacuum tubes without anticoagulant to assess serum concentrations of leptin, ghrelin, adiponectin, cortisol, free T3, free T4, and insulin. After clot retraction, the blood samples were centrifuged at 3,000 rpm for five minutes, and the serum was separated into 0.5 ml aliquots and frozen at −20 °C until the analysis was processed. Serum concentrations of leptin, ghrelin, adiponectin, and insulin were determined via the sandwich ELISA using a specific commercial kit and an ELISA device. Serum concentrations of cortisol, free T3, and free T4 were analyzed using chemiluminescence at the Clinical Analysis Laboratory Hermes Pardini/Veterinary Division.

Hormonal profiles were assessed using samples collected every 72 hours (T0: beginning of inanition; T1: 48 hours after treatment; T2: 144 hours after treatment [end of starvation period]; T3: 72 hours after refeeding and T4: 144 hours after refeeding [immediately before the last surgery]).

The experimental design was completely randomized, with a 4 x 5 factorial scheme, including the two experimental phases (starvation and refeeding). The data were tabulated in an Excel® spreadsheet, and the means were evaluated using the statistical program PROC UNIVARIATE and PROC NPARIWAY at a significance level of 95% (p<0.05). The means were submitted to the PROC UNIVARIATE procedure to assess normality, using the Shapiro-Wilk (SW) test. Aiming at homocesdasty of responses, data with non-parametric distribution were subjected to transformation (Log X + 1). In those cases, in which the variables are associated with a non-parametric distribution, the Kruskal-Wallis tests were used to compare three or more means, and the Wilcoxon test was used to compare two means.

## Results

Mean insulin levels significantly varied over time in each group; moreover, significant differences (p<0.05) were observed between groups at the same time points. In the ENTFL, ENTGL, and PARFL groups, although insulin concentrations increased to levels higher than those recorded at the onset of starvation, values remained within the reference range for the species. However, in the PARGLU group, hyperinsulinemia was detected at the end of the starvation (T2) (Table 1).

**Table 1:** Mean ± standard error of serum insulin concentration (μU/ml) in horses under starvation following exploratory laparotomy, subjected to parenteral fluid therapy (PARFL), enteral fluid therapy with (ENTGL) or without glutamine (ENTFL), and total parenteral nutrition associated with glutamine (PARGL).

**Times**	**Experimental groups**			
	**ENTFL**	**ENTGL**	**PARFL**	**PARGL**
T_0_	0.05 ± 0.02^Cb^	0.39 ± 0.23^Bab^	1.05 ± 0.30^Aa^	0.62 ± 0.28^Bab^
T_1_	1.74 ± 0.08^Ab^	1.74 ± 1.54^ABb^	0.78 ± 0.42^Ab^	3.56 ± 1.13^Aa^
T_2_	0.66 ± 0.02^ABb^	0.86 ± 0.61^Bb^	2.86 ± 0.85^Aa^	5.25 ± 2.63^Aa^
T_3_	0.46 ± 0.31^Bb^	3.12 ± 1.47^Aa^	2.03 ± 1.02^Aa^	0.94 ± 0.50^Bab^
T_4_	1.20 ± 0.19^Aab^	2.24 ± 1.86^Aa^	0.90 ± 0.42^Ab^	1.47 ± 1.03^Bab^

Means followed by different uppercase letters in the column and lowercase letters in the row differ significantly (p<0.05 – Wilcoxon test). Reference value: 0 to 5 μU/ml (Kaneko et al., 2008).

A significant increase in serum glucose concentration was observed in all groups (p<0.05) during the starvation period (T1), which decreased to the baseline in the ENTFL, ENTGL, and PARGL groups after refeeding (Table 2). Only in the PARGL group, serum glucose level remained elevated (p<0.05) even after refeeding, and it was reduced to the baseline 72 hours after the onset of refeeding.

**Table 2:** Mean ± standard error of plasma glucose concentration (mg/dL) in horses under starvation following exploratory laparotomy, subjected to parenteral fluid therapy (PARFL), enteral fluid therapy with (ENTGL) or without glutamine (ENTFL), and total parenteral nutrition associated with glutamine (PARGL).

**Times**	**Experimental groups**			
	**ENTFL**	**ENTGL**	**PARFL**	**PARGL**
T_0_	98 ± 6.16^B^	90.75 ± 2.28 ^B^	99.25 ± 6.88 ^B^	96.00 ± 7.13 ^B^
T_1_	115.5 ± 3.92^A^	108.50 ± 4.63^A^	220.75 ± 98.72 ^A^	143.25 ± 22.76 ^A^
T_2_	105.5 ± 10.87^AB^	98 ± 2.30^AB^	116.75 ± 6.32^AB^	171.00 ± 43.64^A^
T_3_	99.75 ± 2.01^AB^	107.33 ± 4.05^AB^	100.00 ± 9.99^B^	95.33 ± 7.53^B^
T_4_	98 ± 2.0^B^	91.33 ± 5.24^AB^	100.00 ± 7.15^B^	101.33 ± 10.17^AB^

Transformed means followed by different uppercase letters in the column and lowercase letters in the row differ significantly (p<0.05 – Wilcoxon test). Reference value: 75 to 115 mg/dL (Kaneko et al., 2008).

T3 and free T4 measurements revealed no variation in serum glucose levels (p>0.05) between groups or sampling times (Table 3).

**Table 3:** Mean ± standard error of serum T3 (μg/mL) and T4 (μg/dL) levels in horses under starvation after exploratory laparotomy, subjected to parenteral fluid therapy (PARFL), enteral fluid therapy with or without glutamine (ENTGL and ENTFL, respectively), and total parenteral nutrition with glutamine (PARGL).

**Times**	**Experimental groups**			
	**ENTFL**	**ENTGL**	**PARFL**	**PARGL**
**T3 (μg/dl)**				
T_0_	0.57 ± 0.10	0.51 ± 0.14	0.54 ± 0.07	0.60 ± 0.14
T_1_	0.43 ± 0.06	0.27 ± 0.06	0.46 ± 0.05	0.28 ± 0.04
T_2_	0.45 ± 0.16	0.42 ± 0.21	0.46 ± 0.06	0.66 ± 0.21
T_3_	0.32 ± 0.14	0.84 ± 0.30	0.65 ± 0.25	0.86 ± 0.47
T_4_	0.37 ± 0.03	0.48 ± 0.22	0.34 ± 0.06	0.30 ± 0.09
**T4 (μg/dl)**				
T_0_	0.64 ± 0.08	0.69 ± 0.11	0.55 ± 0.12	0.80 ± 0.09
T_1_	0.67 ± 0.08	0.49 ± 0.10	0.54 ± 0.13	0.59 ± 0.18
T_2_	0.71 ± 0.13	0.74 ± 0.20	0.70 ± 0.05	0.71 ± 0.12
T_3_	0.59 ± 0.10	0.65 ± 0.14	0.53 ± 0.10	0.82 ± 0.24
T_4_	0.79 ± 0.08	0.60 ± 0.11	0.57 ± 0.10	0.58 ± 0.18

Means, after transformation, followed by different uppercase letters in the row differ (p<0.05 – Wilcoxon test). Reference values: T3: 0.30 to 1.15 μg/ml; T4: 0.9 to 2.8 μg/dl (Kaneko et al., 2008).

In all groups, an increase in serum cortisol concentrations was observed 72 hours after the onset of food deprivation; however, only the ENTGL group exhibited a significant increase in the cortisol level (Table 4). Additionally, the cortisol level differed (p<0.05) between groups at T3 and T12.

**Table 4:** Mean ± standard error of serum cortisol levels (mg/dL) in horses under starvation after exploratory laparotomy, subjected to parenteral fluid therapy (PARFL), enteral fluid therapy with (ENTGL) or without glutamine (ENTFL), and total parenteral nutrition with glutamine (PARGL).

Times	Experimental groups			
	ENTFL	ENTGL	PARFL	PARGL
T_0_	1.85 ± 0.50	2.77 ± 0.74^B^	2.05 ± 0.50^B^	2.12 ± 0.40
T_1_	2.90 ± 0.38^b^	5.67 ± 0.34^A^	2.80 ± 0.19^ABb^	3.60 ± 0.45^ab^
T_2_	2.97 ± 0.38	3.53 ± 0.98^AB^	2.87 ± 0.44^AB^	3.90 ± 0.70
T_3_	4.00 ± 1.22	2.63 ± 0.43^B^	4.45 ± 1.59^A^	2.96 ± 0.42
T_4_	2.76 ± 1.12^ab^	1.96 ± 0.23^Bb^	5.00 ± 2.17^Aa^	3.20 ± 0.45^ab^

Means, after transformation, followed by different uppercase letters in the row differ (p<0.05 – Wilcoxon test). Reference value: 1.3 to 2.93 mg/dL (Kaneko et al., 2008).

Serum ghrelin did not significantly differ between groups at any evaluated time points (p>0.05). Only the PARGL group exhibited an increase in ghrelin concentration (p<0.05) after food deprivation and a decrease in serum ghrelin levels after refeeding (p<0.05) (Table 5).

**Table 5:** Mean values ± standard error of serum ghrelin concentration (ng/dL) in horses under starvation after exploratory laparotomy, submitted to parenteral fluid therapy (PARFL), enteral fluid therapy with (ENTGL) or without glutamine (ENTFL), and total parenteral nutrition supplemented with glutamine (PARGL).

**Times**	**Experimental groups**			
	**ENTFL**	**ENTGL**	**PARFL**	**PARGL**
T_0_	0.018 ± 0.002	0.014 ± 0.003	0.021 ± 0.003	0.021 ± 0.001^A^
T_1_	0.017 ± 0.002	0.018 ± 0.002	0.016 ± 0.001	0.016 ±0.0007 ^B^
T_2_	0.014 ± 0.003	0.018 ± 0.005	0.023 ± 0.003	0.019 ± 0.002^AB^
T_3_	0.019 ± 0.001	0.017 ± 0.003	0.022 ± 0.003	0.011 ± 0.005^B^
T_4_	0.023 ± 0.011	0.020 ± 0.001	0.022 ± 0.005	0.011 ± 0.002^B^

Means, after transformation, followed by different uppercase letters in the column and lowercase letters in the row differ (p<0.05 – Wilcoxon test).

The evaluation of serum adiponectin concentrations revealed no significant difference (p>0.05) in the ENTFL, ENTGL, and PARGL groups; However, in the PARFL group, serum concentrations increased (p<0.05) after food deprivation and decreased (p<0.05) after the refeeding period (Table 6).

**Table 6:** Mean values ± standard error of serum adiponectin concentration (ng/mL) in horses under starvation after exploratory laparotomy, treated with parenteral fluid therapy (PARFL), enteral fluid therapy with (ENTGL) or without glutamine (ENTFL), and total parenteral nutrition with glutamine (PARGL).

**Times**	**Experimental groups**			
	**ENTFL**	**ENTGL**	**PARFL**	**PARGL**
T_0_	0.028 ± 0.014	0.022 ± 0.008^A^	0.011 ± 0.006^B^	0.03 ±0.02
T_1_	0.038 ± 0.011	0.033 ± 0.006^A^	0.039 ± 0.016^AB^	0.050 ± 0.01
T_2_	0.044 ± 0.02	0.022 ± 0.0^A^	0.061 ± 0.016^A^	0.034 ± 0.014
T_3_	0.045 ± 0.02	0.037 ± 0.007^A^	0.04 ± 0.01^AB^	0.015 ± 0.007
T_4_	0.059 ± 0.03^a^	0.00^Bb^	0.034 ± 0.014^Aba^	0.118 ± 0.09^a^

Means, after transformation, followed by different uppercase letters in the column and lowercase letters in the row differ (p<0.05 – Wilcoxon test).

Furthermore, serum leptin level was reduced (p<0.05) after food deprivation in the ENTGL and PARGL groups; moreover, refeeding induced an increase in its concentration (Table 7).

**Table 7:** Mean values ± standard error of serum leptin concentration (ng/mL) in horses under starvation after exploratory laparotomy, treated with parenteral fluid therapy (PARFL), enteral fluid therapy with (ENTGL) or without glutamine (ENTFL), and total parenteral nutrition with glutamine (PARGL).

**Times**	**Experimental groups**			
	**ENTFL**	**ENTGL**	**PARFL**	**PARGL**
T_0_	0.18 ± 0.016	0.14 ± 0.02^AB^	0.16 ± 0.003	0.19 ± 0.005^A^
T_1_	0.15 ± 0.01^ab^	0.09± 0.03^Bb^	0.18 ± 0.01^a^	0.15 ± 0.01^ABab^
T_2_	0.14 ± 0.01	0.17± 0.01 ^AB^	0.16 ± 0.01	0.12 ± 0.02^B^
T_3_	0.16 ± 0.01	0.13 ± 0.024^AB^	0.16 ± 0.01	0.13 ± 0.02^AB^
T_4_	0.14 ± 0.01	0.19 ± 0.02^A^	0.15 ± 0.02	0.17 ± 0.02^AB^

Means, after transformation, followed by different uppercase letters in the column and lowercase letters in the row differ (p<0.05 – Wilcoxon test). Reference values: 3,47ng/ml RIA (Radin et al., 2009).

## Discussion

A main mechanism underlying the adaptation to prolonged starvation periods involves the alteration in hormonal balance, particularly the decrease in insulin production (Geor, 2013). However, in the present study, a decrease in serum insulin concentrations was not observed; in all groups, nutritional support provided via nasogastric or parenteral routes enhanced blood glucose levels, which led to an increase in serum insulin concentration.

In the ENTFL group, serum glucose peaks were accompanied by insulin peaks, except at T4 (after the refeeding period), when a peak in serum insulin concentration occurred without a concomitant increase in plasma glucose. Nevertheless, serum insulin concentration remained within the species-specific reference range (Kaneko et al., 2008).

In the ENTGL group, a peak in plasma glucose was detected 72 hours after the initiation of refeeding, which was accompanied by a rise in serum insulin. Ferrufino et al. (1996) reported the average time for mucosal recovery to be 3–6 days. Hence, all animals in the ENTGL group likely experienced intestinal mucosal recovery between the sampling periods, which resulted in a glycemic peak.

Furthermore, the gradual refeeding process was potentially associated with the glycemic peak in this group. The diet was gradually reintroduced over three days, and by the fourth day, the animals had resumed their feeding level as recorded during the pre-experimental adaptation period. This readaptation period was probably insufficient for these animals, causing the diet to have a high glycemic index.

In the PARFL group, serum insulin profile did not follow the increase in serum glucose recorded 72 hours after the onset of food deprivation (T3). According to Ralston (2002), blood glucose and insulin concentrations are influenced by various factors, and their concentrations in the systemic circulation at a time point are associated wth the balance between the rates of entry and removal of glucose from the circulation (Nogueira et al., 2003; Kaneko et al., 2008). For instance, diurnal variations in cortisol, which can antagonize insulin, are a major factor (Canali & Kruel., 2001). In this study, we detected an increase in serum cortisol concentrations 72 hours after the onset of food deprivation, which may explain the unpredictable variation in serum insulin concentration at this time.

In the PARGL group, plasma glucose peaks were accompanied by corresponding peaks in insulin secretion, as expected. Nutritional support provided via the enteral route (ENTFL and ENTGL) promoted glycemia as that observed in the two groups that received nutritional support via the parenteral route (PARFL and PARGL). Therefore, despite the reduction in absorptive surface by approximately 1.8 times and 1.2 times in the ENTFL and ENTGL groups, respectively, the intestinal mucosa was able to maintain its absorptive capacity for glucose supplied enterally, even under atrophic conditions.

Some studies demonstrated alterations in T3 and T4 concentrations during starvation or food deprivation (Hennemann & Krenning, 2007; Powell et al., 2000). Equine models of short-term starvation (48 hours) exhibited significant reductions in T3 and T4 concentrations (Christensen et al., 1997). Despite a longer period of food deprivation included in our study, no changes in T3 and T4 concentrations were detected.

Food deprivation reduces insulin and increases serum glucagon concentrations. These changes exhibit glycogenolytic and lipolytic effects, and increase the concentrations of free fatty acids in the blood (Campbell, 2004). Additionally, a decrease in thyroid hormone synthesis (T3) helps reduce metabolic rate and daily energy requirements. However, despite this important role in thyroid hormone metabolism, we detected no alterations across the time points and groups.

This result can be explained by the blood glucose levels observed in the animals during the food deprivation period, as all groups showed an increase in serum glucose, which probably prevented the decrease in circulating T3 and T4 levels in this experimental model.

Food intake may modulate the hypothalamic-pituitary-adrenal (HPA) axis response to stress and glucocorticoids, especially cortisol, contributing to the neuroendocrine control of food intake and energy expenditure. Although studies involving food restriction in rats indicated that food is the primary synchronizer of the HPA axis, the association between this axis and feeding in both humans (Douyon & Schteingart, 2002) and horses remains unclear.

However, an increase in circulating cortisol is expected to enhance lipolysis, gluconeogenesis, and protein catabolism in animals subjected to food deprivation (Kaneko et al., 2008), which facilitates nutrient mobilization. In this study, the cortisol (p<0.05) level was increased 72 hours after the onset of food deprivation, possibly to increase gluconeogenesis (Guyton & Hall, 2006) and antagonize the rise in circulating insulin in all groups after 72 hours of food deprivation; this alteration was significant in the ENTFLU and PARGLU groups.

In contrast to our study outcomes, Sticker et al. (1995) observed a reduction in plasma cortisol concentration in a model of energy restriction in mares. Similarly, Glade et al. (1984) reported a decrease in cortisol concentration in foals subjected to restricted energy intake.

Guimarães et al. (2007) found that fasting did not affect serum cortisol concentrations in dogs immediately before inhalation anesthesia, which was consistent with another report (Reimers et al., 1986), which reflected that basal cortisol concentrations are not affected by a 36-hour fast in dogs. However, those studies did not demonstrate cortisol levels over prolonged periods. Therefore, based on the present findings, we suggest that cortisol assessment beyond 36 hours should be considered to evaluate equine patients under food deprivation or starvation.

In addition to the difference detected 72 hours after food deprivation, further differences (p<0.05) were observed 120 hours after the onset of refeeding. This is attributed to the increase in circulating cortisol concentrations in the PARFLU group at this point. This increase may be related, particularly in this group, to the removal of the long-term catheter maintained throughout the experimental period. Although no inflammation was observed around the double-lumen catheter used in this study, the animals exhibited restlessness and discomfort during their handling, which potentially induced a stress response.

Ghrelin is mainly secreted by gastric cells, with a small portion synthesized in other sites, including the hypothalamus, heart, kidneys, lungs, and duodenum (Mota & Zanesco, 2007). Thus, intestinal atrophy may have proportionally compromised ghrelin secretion because, in addition to the atrophy of the small intestine observed in this study, the effects of food deprivation on the gastric mucosa and the synthesis and secretion of this hormone remain unknown.

Furthermore, circulating ghrelin levels increase during starvation and hypoglycemic states (Romero & Zanesco, 2006). However, we detected no increase in circulating ghrelin levels (p>0.05) in the ENTFL, ENTGL, and PARFL groups after food deprivation, which is possibly attributed to the increased serum glucose in response to nutritional support in these animals, which probably antagonized ghrelin release by gastric cells.

Only the PARGL group exhibited a significant increase (p<0.05) in ghrelin concentration during food deprivation and a decrease (p<0.05) after refeeding. Erdmann et al. (2004) reported that protein or amino acid-rich nutritional support, such as total parenteral nutrition supplemented with glutamine, can increase serum ghrelin concentrations. However, in this study, the hyperglycemic state potentially prevented the manifestation of this response.

Long-term energy manipulations (over three months), such as energy restriction, increase adiponectin gene expression and its levels in the circulatory system (Imbeault et al., 2004). However, this study, which included a short period of food restriction, revealed no hypoglycemia in the tested groups, which could have led to a decrease in serum adiponectin concentration.

Fasting or refeeding can rapidly regulate adiponectin (Calvani et al., 2004). However, in horses, adiponectin concentration does not fluctuate in a circadian pattern in response to feeding (Radin et al., 2009), which is consistent with the present findings.

In the PARFL group, the increase in circulating adiponectin (p<0.05) coincided with a non-significant (p>0.05) increase in insulin. Although the mechanisms underlying this association are complex and context-dependent, current evidence supports a direct influence of adiponectin on pancreatic β-cells. Adiponectin enhances insulin sensitivity in peripheral tissues and promotes insulin gene expression and secretion by activating intracellular signaling pathways, including Akt and ERK in β-cells (Wijesekara et al., 2010). Additionally, it confers cytoprotective effects by reducing apoptosis under metabolic stress. These actions may be associated with the trend observed in this study.

Serum leptin levels are influenced by nutritional, hormonal, and physiological factors, such as fasting and exercise, potentially inhibiting gene expression and a subsequent drop in the plasma level of leptin (Romero & Zanesco, 2006), which aligns with the data revealed for the ENTGL and PARGL groups in this study. Furthermore, when serum leptin concentration was assessed over time, a reduction (p<0.05) was observed 72 hours (T3) after initiating the food deprivation in the ENTGL group than in the other groups. Leptin secretion by adipocytes is affected by various hormones, including insulin (Ramsay & White, 2000) and glucocorticoids (Wang et al., 2002). Our study revealed an increase in both insulin (p<0.05) and cortisol (p<0.05) 72 hours after the onset of food deprivation, which may explain the change observed in serum leptin levels.

Previous studies reflected that food-deprived horses (Buff et al., 2005; Piccione et al., 2004) exhibit a reduction in plasma leptin concentration compared to animals fed ad libitum. Similar homogeneous changes were not observed in our analyses, which is potentially attributed to the increase in serum glucose, caused by the nutritional support, during food deprivation in animals. Hence, serum glucose possibly served as a modulator, preventing a significant increase in serum leptin concentrations in all tested groups.

The association between nutritional support, hormonal profile, and glycemic stability suggests that both the tested routes of administration and the nutritional composition directly influence the endocrine response. Differences between enteral and parenteral groups, as well as the presence or absence of glutamine, revealed subtle but potential clinical effects on the secretion of hormones involved in energy homeostasis.

Despite the significance of the findings, this study has limitations that should be considered while interpreting the outcomes and designing future investigations. The small number of animals per tested group and the short hormonal monitoring period may have limited the detection of late endocrine changes, particularly those associated with prolonged refeeding phases and potential rebound effects in energy metabolism. Additionally, all experimental groups received some form of nutritional support, precluding the isolated assessment of endocrine repercussions associated with absolute starvation, which complicated the characterization of the metabolic impact of food restriction alone.

Moreover, limited hormonal profiles were evaluated. Although key hormones such as cortisol, leptin, ghrelin, adiponectin, and thyroid hormones were included, analyses of additional markers such as glucagon, catecholamines, growth hormone (GH), and insulin-like growth factor 1 (IGF-1) could provide a more comprehensive overview of the neuroendocrine response to nutritional stress. The absence of these assays constrains the understanding of the interactions among different endocrine axes involved in substrate metabolism, especially during the transition from fasting to refeeding.

Moreover, potential confounding factors related to experimental stress, such as physical restraint, frequent handling, and invasive interventions, must be considered, as they can influence serum cortisol levels and affect other hormones regulated by this axis.

Therefore, future studies should prioritize extending the postoperative follow-up period to assess delayed hormonal responses and potential metabolic complications associated with refeeding. The inclusion of experimental groups subjected to absolute fasting (without nutritional support) and comparisons between different routes and compositions of nutritional support (variable glucose, lipid, and specific amino acid contents) can further clarify the regulatory mechanisms involved in endocrine adaptation.

This study advances current knowledge regarding the endocrine effects of nutritional support in equine models during food deprivation and refeeding. Nevertheless, the results underscore several critical gaps that warrant further investigation. Future studies involving larger cohorts and more frequent hormonal sampling intervals (<24 h) can elucidate acute endocrine fluctuations and clarify the chronobiological responses to nutritional stress. The inclusion of experimental groups subjected to complete fasting, with no nutritional support, may reveal the isolated effects of caloric deprivation on the hypothalamic-pituitary-adrenal axis and gut-derived hormonal responses. Moreover, expanding the endocrine panel to include additional markers such as glucagon, IGF-1, catecholamines, and acylated ghrelin, as well as pro- and anti-inflammatory cytokines, can provide a more comprehensive view of the neuroimmune-endocrine adaptations involved. Finally, exploring the role of the gut microbiota and epithelial barrier integrity during the fasting–refeeding transition may reveal novel interactions among energy metabolism, mucosal immunity, and endocrine function in large animals.

## Conclusions

In conclusion, the results reveal that the nutrient composition and route of administration of nutritional therapy significantly influenced the hormonal response in horses after exploratory laparotomy. Different combinations, especially the addition of glutamine and the selection of total parenteral nutrition or enteral therapy, distinctly modulated hormone levels, reflecting important metabolic adaptations in the postoperative period. These results highlight the importance of personalized nutritional therapy to effectively achieve clinical recovery, involving the promotion of a favorable hormonal balance to improve the overall condition and recovery of animals undergoing complex surgical procedures.
